# Draft genome sequence of *Sanguibacter* species strain 25GB23B1, cultivated from arctic surface seawater

**DOI:** 10.1128/mra.00868-24

**Published:** 2024-10-04

**Authors:** Kiana Mitchell, Grace Busch, Indu Sharma

**Affiliations:** 1 Department of Biological Sciences, Hampton University, Hampton, Virginia, USA; DOE Joint Genome Institute, Berkeley, California, USA

**Keywords:** laminarin

## Abstract

We report a draft genome sequence for *Sanguibacter* species strain 25GB23B1, isolated from arctic surface seawater off the coast of Alaska. The whole genome sequence will provide knowledge of the bacteria’s relationship to its environment and possibly a new species of *Sanguibacter*.

## ANNOUNCEMENT

*Sanguibacter* is a genus of Gram-positive, coryneform bacteria under the family of *Cellulomonadaceae*. Members of this genus have been found in marine environments, specifically in coastal sediment from the Eastern China Sea (1) and in ice in Antarctica (2). The *Sanguibacter* strain of this study was isolated from arctic seawater accessed 1 m below the water’s surface through drilling holes with an ice auger near North Slope Borough, Alaska on 22 May 2023 at the coordinates 70° 27′ 30.0″ N, 148° 25′ 06.2″ W and named 25GB23B1.

An enrichment was performed with a medium modified from Unfried et al. (3) [342.2 mM NaCl, 14.8 mM MgCl_2_·6H_2_O, 1.0 mM CaCl_2_·2H_2_O, 6.71 mM KCl, 100 mg casamino acids, 100 mg peptone, 20 mM MOPS (pH 8.0), 10 mM NH_4_Cl, 10 mM KH_2_PO_4_, 1 mM Na_2_SO_4_, 1 mM trace metals, and 0.5% laminarin]. The culture was incubated for 7 days at 30°C on an orbital shaker (100 rpm shaking). From this enrichment, 100 µL was plated on an artificial seawater complete medium [342.2 mM NaCl, 14.8 mM MgCl_2_·6H_2_O, 1.0 mM CaCl_2_·2H_2_O, 6.71 mM KCl, 5 g Bacto-tryptone, 1 g yeast extract, 20 mM MOPS (pH 8.0), and 0.3% glycerol] and agar at a final concentration of 1.5%. The colony was selected for its bright yellow color and streaked for isolation on the same artificial seawater media.

For whole-genome sequencing, DNA was extracted from 2 mL culture of microbial isolate *Sanguibacter* sp. 25GB23B1 using the Zymo Quick-DNA Fungal/Bacterial Miniprep Kit (Zymo). The sample was received and quantified using an Invitrogen Qubit 4 Fluorometer and 1× dsDNA High Sensitivity Assay Kit (ThermoFisher Scientific). The genomic library was prepared using DNA extracts and the Nextera XT DNA Library Preparation Kit (Illumina) according to the manufacturer’s protocol. The library was quality checked using an Agilent 2100 Bioanalyzer and DNA High Sensitivity Kit and then pooled in an equimolar ratio. The pool was gel purified using a 2% agarose gel and the Qiagen QIAquick Gel Extraction Kit (Qiagen). Following purification, the pool was sequenced on an Illumina NextSeq 550 instrument using a Mid-Output version 2.5 chemistry 300-cycle kit to produce 2 × 150-bp reads. Amplification of the 16S rRNA gene using the primers 533F and 1100R and sequencing confirmed that the isolate is a member of the genus *Sanguibacter*.

**Fig 1:**
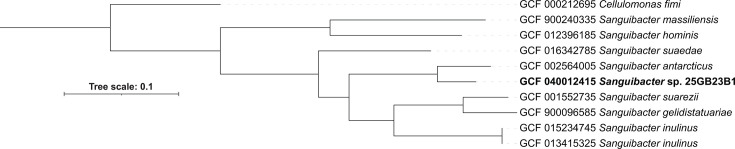
Phylogenomic tree for *Sanguibacter* sp. isolate 25GB23B1 based on 74 unique single-copy core genes depicting the phylogenomic position relative to other *Sanguibacter* sp. *Cellulomonas fimi* was used as an outlier.

The raw genomic data were analyzed using FastQC (4) and found to have a total number of 7,418,186 raw sequences with a length of 151 bp. Trimmomatic version 0.39 (5) was used for quality filtering and trimming the raw Illumina reads using the following parameters: LEADING:3 TRAILING:3 SLIDINGWINDOW:4:20 MINLEN:60. A combined 3,513,311 individual forward and reverse reads were retained, which were used to generate the genome assembly. For all the following programs, default settings were used. Assembly was performed from the trimmed reads using SPAdes version 3.13.0 (6), and assembly summary statistics were generated with QUAST version 5.0.2 (7). The draft genome’s total length is 4,303,413 bp, composed of 66 contigs (*N*_₅₀_: 96,636 bp, *L*_₅₀_: 14), with an average GC content of 70.68% and an average genome coverage of ~123-fold. The genome was annotated by Rapid Prokaryotic Genome Annotation (8) and found to contain 3,972 protein-coding sequences, 3 complete rRNA genes, 54 tRNA genes, and 1 tmRNA gene. A phylogenomic tree was created using the Interactive Tree of Life (9) and GToTree program (10) with *Cellulomonas fimi* as an outlier for comparison (Fig. 1). The average nucleotide identity, calculated using pyANI version 0.1.2 (11) default settings, between 25GB23B1 and *Sanguibacter antarcticus* (GCF_002564005.1) was 84.57%, with ~68% of the two genomes aligning. As a genome needs to share 97% or more DNA with another genome to be considered the same species, the *Sanguibacter* strain 25GB23B1 ascribes to one of the criteria of being a new species of *Sanguibacter* (12).

## Data Availability

This whole-genome shotgun genome assembly has been deposited at NCBI GenBank under accession number JBDYKL000000000.1. The BioProject accession number is PRJNA1113969 and the BioSample accession number is SAMN41472747. Raw sequencing reads were deposited in NCBI’s Sequence Read Archive under accession number SRX25616898.
